# Non-Arteritic Anterior Ischemic Optic Neuropathy (NAION) Following a Hypovolemic Episode of Gastric Bleeding

**DOI:** 10.7759/cureus.11627

**Published:** 2020-11-22

**Authors:** Evangelos Gkoumas, Georgios Bontzos, Tina Xirou, Efterpi Chatzispasou, Stamatina Kabanarou

**Affiliations:** 1 Ophthalmology, Korgialenio-Benakio General Hospital, Athens, GRC

**Keywords:** anterior ischemic optic neuropathy, optic nerve, hypovolemia, hypoperfusion

## Abstract

Non-arteritic anterior ischemic optic neuropathy (NAION) is a rare complication following acute bleeding. Patients present with varying vision loss and visual field defects. NAION is more commonly developed in patients with systemic disorders that may affect normal blood flow such as hypertension and diabetes. In this case, we report a 54-year-old man who complained of vision blurring following an episode of acute gastric bleeding. This report aims to review the pathology of this condition and present the findings of newer non-invasive imaging modalities of the vascular layers of the posterior pole of the eye like optical coherence tomography angiography (OCTA), which facilitates the proper diagnosis and prognosis of such cases. Finally, we present the management options for this patient with antiplatelet treatment.

## Introduction

Ischemic optic neuropathy is a severe sight-threatening disorder of the optic nerve. It is the most common acute neuropathy in patients >50 years, with an estimated annual incidence in the United Stated of 2.3-10.2 cases per 100,000 population [1]. It is typically classified as posterior ischemic optic neuropathy and anterior ischemic optic neuropathy (AION). AION is caused by ischemia of the anterior part of the optic, supplied by short ciliary arteries. Pathogenetically, AION can be either arteritic (AAION) due to giant cell arteritis (10-15%) or non-arteritic (NAION), which includes the rest of the causes [2]. NAION typically presents with painless visual diminution over hours or days [3]. Differential diagnosis includes other vascular conditions like retinal and veins occlusion, optic neuritis, or acute optic nerve compression. A detailed history and clinical examination usually reveal the underlying cause of vision loss. NAION has been associated with risk factors for vascular occlusion like systemic hypertension, diabetes, hyperlipidemia, and smoking [4-7]. Regarding cellular mechanisms, advances in understanding ischemic central nervous system (CNS) damage have shed light upon the pathogenesis and neural damage in NAION [8]. Secondary neuronal damage in the adjacent tissues of the hypoperfusion can develop as a result of a toxic environment, specifically induced by glutamate, intracellular calcium influx, and reactive oxygen species [9].

## Case presentation

A 54-year-old man presented to the ophthalmology clinic with a four-day history of blurred vision in his right eye (OD). The patient had an event of acute gastric bleeding due to duodenal ulcer perforation five days earlier. He did not have previous intraocular surgery, ocular inflammation, or another eye disease. He reported a history of a lazy left eye (OS) and a red-green color deficiency history from childhood. The patient’s medical history included hypertension and occasional use of non-steroidal anti-inflammatory drugs (NSAIDs) for lower back pain.

At presentation, his best-corrected visual acuity (BCVA) was 20/65 OD and 20/100 OS. Intraocular pressure was 10 mmHg in both eyes, using Goldmann applanation tonometry. Pupils were symmetric and light and near pupillary reflexes were normal. Confrontation field testing revealed an upper hemianopic defect OD. His anterior segment examination was insignificant. Dilated fundus examination of his OD revealed optic disc edema, peripapillary flame-shaped hemorrhages, and cotton wool spots nasally from the fovea. In his OS, there were a few flame-shaped hemorrhages at the peripapillary area. Retinal vessels showed early signs of hypertensive retinopathy with arteriolar constriction and spots of arteriovenous nicking in both eyes.

**Figure 1:**
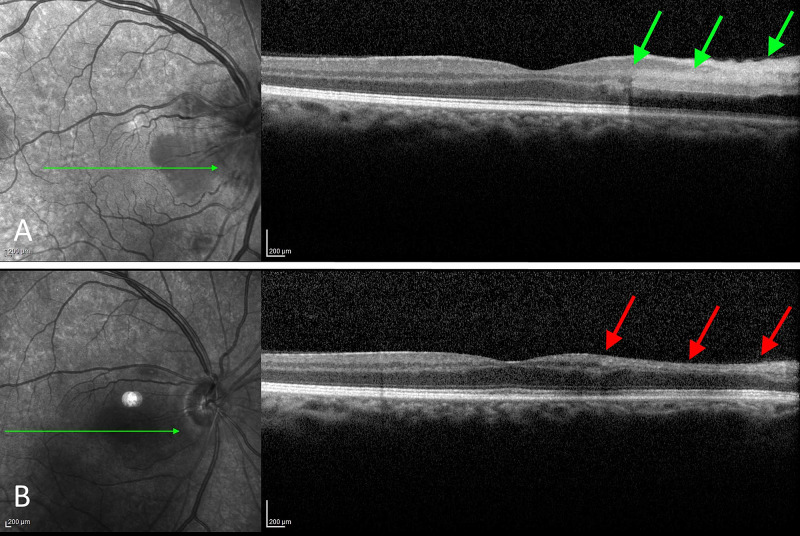
OCT of the right eye upon presentation and one month later A. OCT of the posterior pole reveals an area of increased reflectivity and thickening of the inner retina nasal to the fovea (green arrows). The optic disc appears edematous in the infrared image. B. One month later, resolution of the edema and associated atrophy of the involved area was observed (red arrows). OCT: optical coherence tomography

Optical coherence tomography (OCT), shown in Figure 1, revealed an area of increased reflectivity and thickening of the inner layers nasally of the fovea in his OD (Figure 1A).

**Figure 2:**
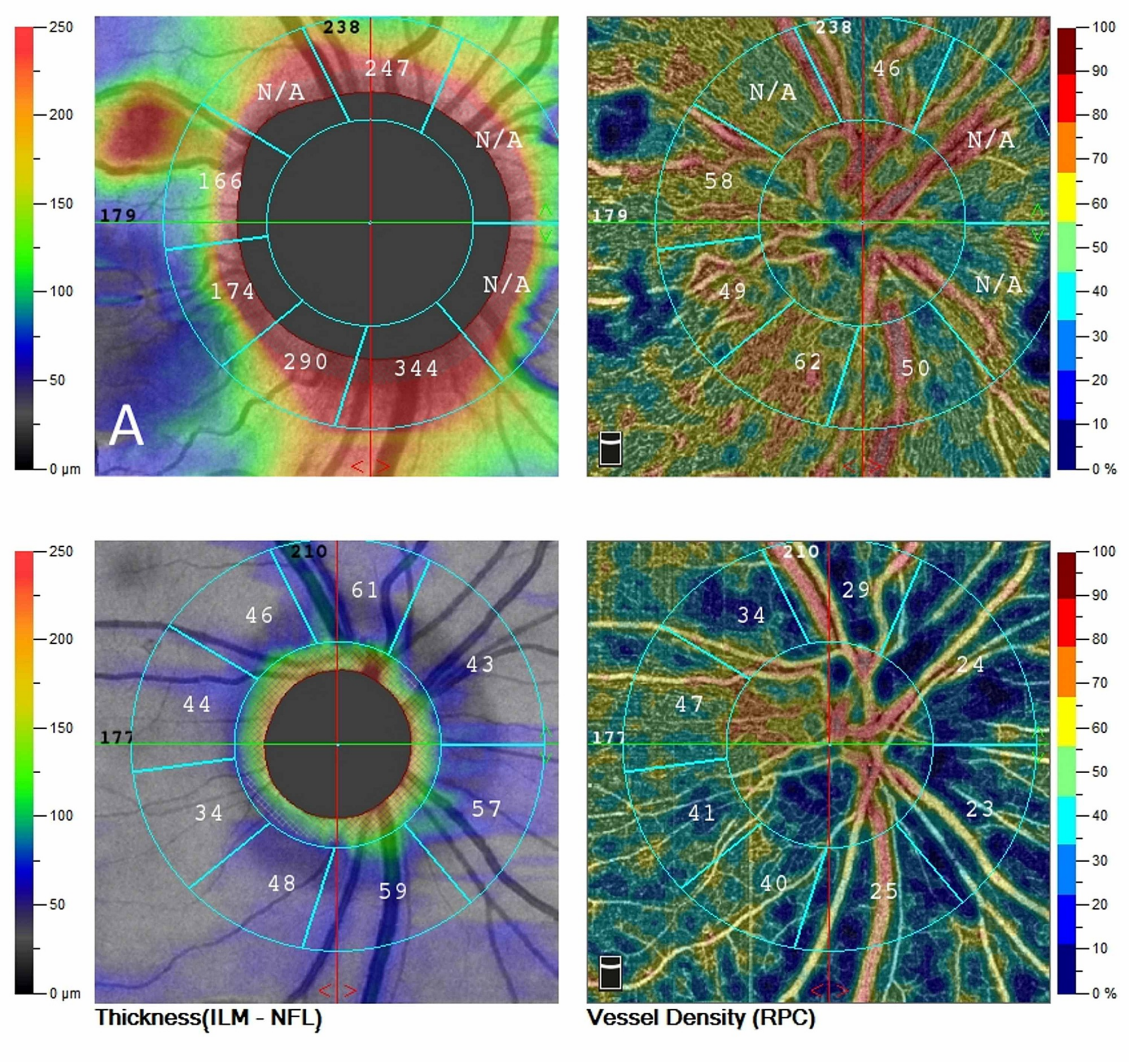
OCT angiography of the optic disc A. Upon presentation, diffuse edema with normal vascular perfusion was observed. B. A month later, optic disc atrophy was established with associated thinning of the peripapillary fiber layer. Extensive areas of reduced perfusion are observed in the peripapillary area. Flow remains within normal limits temporal to the optic disc. OCT: optical coherence tomography

OCT was an unremarkable OS. OCT angiography (OCTA) of the optic nerve, shown in Figure 2, revealed an increased thickness of the superficial layer indicative of an edematous disc but with normal perfusion (Figure 2A).

**Figure 3:**
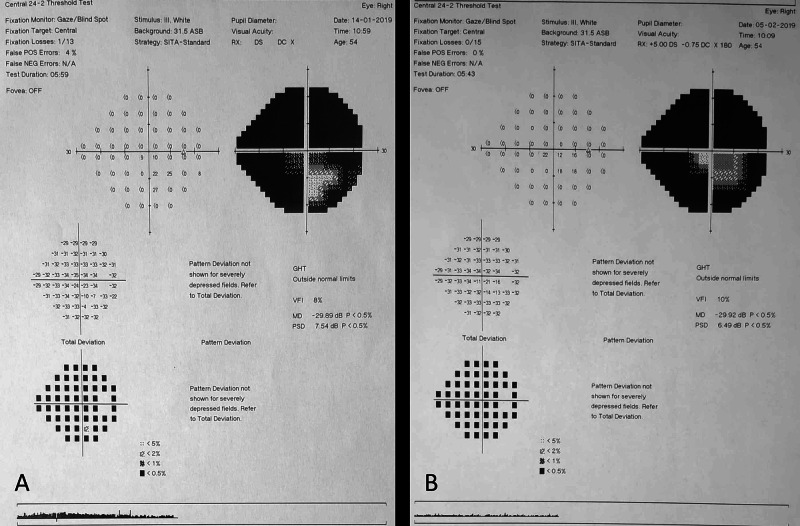
Visual field testing with Humphrey 24-2 A. At baseline, perimetry shows generalized depression of the visual field OD, with a central island of vision. B. Defects remained stable one month later.

Standard automated perimetry, using the Humphrey 24-2 field test (Figure 3), revealed generalized depression OD, with an area of relative defect in the lower field while OS was normal (Figure 3A).

During his hospitalization in the internal medicine department, a series of laboratory investigations were undertaken. The erythrocyte sedimentation rate was within normal limits but complete blood count revealed significantly low values of hematocrit and hemoglobin (Hct: 15.1% and Hb: 4.9 g/dL). Blood transfusion was immediately performed and tests were normalized after 12 hours (Hct: 27.4% and Hb: 9.1 g/dL). Further workup, including neuroimaging and inflammatory markers, ruled out compressive, inflammatory, or infiltrative disease causes.

Considering the above clinical, laboratory, and imaging findings, the diagnosis of non-arteritic anterior ischemic optic neuropathy (NAION) was established, which was attributed to the acute loss of blood volume. After extensive discussion with the patient’s treating physician regarding possible complications of antiplatelet treatment, the patient was administered acetylsalicylic acid (100 mg) on a daily basis and was monitored for 10 months following this episode.

One month later, the patient was re-examined. His BCVA was mildly improved to 20/50 in the right eye but remained stable in the left eye. Fundus examination revealed resolution of the optic disc edema OD with the establishment of disc pallor to its temporal margin. Examination of his OS was unremarkable. OCT revealed significant thinning of the inner retina temporally of the macula (Figure 1B) while OCTA revealed areas of peripapillary hypoperfusion OD, nasally (Figure 2B). Automated Humphrey perimetry defects remained stables compared to his previous examination (Figure 3B). We observed that the area of preserved functionality corresponded to the remaining healthy vasculature temporally of the optic nerve. Further follow-up of the patient, for nine months, showed a stable condition with minimal visual recovery and persistent areas of reduced vasculature of the optic disc in OCTA.

## Discussion

NAION following acute blood loss has been described after surgical procedures, most commonly of the gastrointestinal (GI) tract [10-11]. In most patients, vision loss does not improve [10]. Treatment is primarily focused on the prompt restoration of blood volume and correction of anemia, although salvage of vision following such acute changes is difficult [12]. Nevertheless, partial visual recovery has been reported in some patients [12]. With a similar pathology, fluctuations in blood pressure, for instance, in patients with renal failure undergoing dialysis, may result in NAION [13-14].

In our described case, the diagnosis was based upon the patient’s history of an acute hypovolemic episode and the ophthalmic examinations, including fundoscopy, visual field testing, and imaging of the retinal layers (OCT) and vessel density (OCTA) of the posterior pole of the eye. The optic disc edema in our case was resolved within a month. However, an extensive visual field defect remained, with peripapillary thinning of the retinal nerve fibers as demonstrated in OCT. Visual field depression corresponds to the peripapillary areas of reduced vascular perfusion. Although this finding might not be universal, it suggests that OCTA could predict the areas of vision loss in cases of ischemic optic neuropathy. More research is needed, however, to expand these findings in clinical practice, especially for atypical cases.

Currently, there is no standard effective treatment for NAION. Some evidence suggests that there is a higher proportion of patients who may benefit in terms of visual acuity following the administration of antiplatelet therapy [15]. The management of this case was challenging since the administration of antiplatelet treatment had to be weighed against the risk of further GI bleeding. Administration of antiplatelet therapy has been shown to reduce the incidence of NAION in the fellow eye [16], although the beneficial long-term effects remain unproven. Expert recommendations suggest the daily use of aspirin after an initial episode, for decreasing the stroke and myocardial infarction risk in susceptible groups [17-18]. Further treatment options might include oral or intravenous (IV) corticosteroids with a view to preserving nerve damage from secondary inflammation [19], although there is no evidence for better BCVA outcomes. In 2018, a randomized controlled trial showed that despite the faster resolution of optic disc edema, these structural changes were insignificant [20].

## Conclusions

In summary, patients reporting vision loss after acute massive bleeding should be investigated for NAION. OCTA allows the in-vivo observation and monitoring of retinal and optic nerve vascularization. Antithrombotic therapy may be administered with caution and the regular monitoring of the patient’s systemic condition.
